# Supplementary material for: Increasing temperature can modify the effect of straw mulching on soil C fractions, soil respiration, and microbial community composition
Source: PLoS One. 2020 Aug 11;15(8):e0237245. doi: 10.1371/journal.pone.0237245 (PMC7418978; doi:10.1371/journal.pone.0237245)
Supplement: S1 Fig — CK: no mulching; SM: straw mulching. (PDF) [file pone.0237245.s001.pdf]

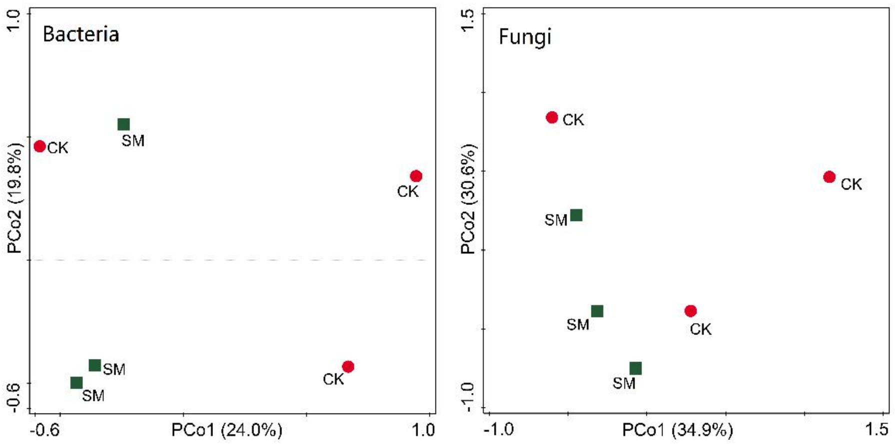

S1 Fig. Principal coordinates analysis (PCoA) of soil microbial community composition under straw mulching and no mulching before incubation based on Bray-Curtis distances.

CK: no mulching; SM: straw mulching.
