# Supplementary material for: Increasing temperature can modify the effect of straw mulching on soil C fractions, soil respiration, and microbial community composition
Source: PLoS One. 2020 Aug 11;15(8):e0237245. doi: 10.1371/journal.pone.0237245 (PMC7418978; doi:10.1371/journal.pone.0237245)
Supplement: S1 Table — (a) CK: no mulching; SM: straw mulching. (b) Different lowercase letters indicate significant difference among different mulching methods. (PDF) [file pone.0237245.s002.pdf]

S1 Table. Influence of straw mulching and temperature on soil carbon fractions after short-term incubation

Incubation temperature (°C)	Treatment	SOC	PCM	MBC
15	CK[a]	8.31b	339b	420a
	SM	8.92a	381a	393a
25	CK	8.20b	328b	371a
	SM	8.82a	398a	346a
35	CK	8.08b	310b	346a
	SM	8.68a	343a	341a

a CK: no mulching; SM: straw mulching

b Different lowercase letters indicate significant difference among different mulching methods
