# Supplementary material for: Increasing temperature can modify the effect of straw mulching on soil C fractions, soil respiration, and microbial community composition
Source: PLoS One. 2020 Aug 11;15(8):e0237245. doi: 10.1371/journal.pone.0237245 (PMC7418978; doi:10.1371/journal.pone.0237245)
Supplement: S2 Table — (a) CK: no mulching; SM: straw mulching. (b) Different lowercase letters indicate significant difference among different mulching methods or incubation temperatures. (c) NS, not significant. ***Significant at P≤0.001; **significant at P≤0.01; *significant at P≤0.05. (PDF) [file pone.0237245.s003.pdf]

S2 Table. Influence of straw mulching and temperature on soil bacterial phyla (%) after short-term incubation

Mulching method[a]	Incubation temperature (°C)	*Proteobacteria*	*Actinobacteria*	*Chloroflexi*	*Acidobacteria*	*Gemmatimonadetes*	*Nitrospirae*	*Planctomycetes*	*Bacteroidetes*
CK		28.4b[b]	20.2a	15.8a	14.2b	8.78a	3.98b	3.63b	1.26b
SM		31.7a	14.4b	13.8b	15.5a	8.59a	5.54a	4.90a	1.69a
	15	28.6a	16.9a	16.0a	16.1a	8.59ab	4.73a	4.30a	1.70a
	25	29.6a	18.0a	15.5a	14.7ab	7.94b	5.15a	4.22a	1.43b
	35	32.0a	16.9a	12.9b	13.8b	9.52a	4.40a	4.28a	1.29b
<u>Significance</u>									
Treatment (T)		*	***	**	*	NS	**	*	***
Temperature (ST)		NS[c]	NS	**	*	*	NS	NS	*
T×ST		NS	NS	NS	NS	NS	NS	NS	NS

a CK: no mulching; SM: straw mulching

b Different lowercase letters indicate significant difference among different mulching methods or incubation temperatures

c NS, not significant.

***significant at $P\leq0.001$; **Significant at $P\leq0.01$; *significant at $P\leq0.05$.
