# Supplementary material for: Increasing temperature can modify the effect of straw mulching on soil C fractions, soil respiration, and microbial community composition
Source: PLoS One. 2020 Aug 11;15(8):e0237245. doi: 10.1371/journal.pone.0237245 (PMC7418978; doi:10.1371/journal.pone.0237245)
Supplement: S3 Table — (a) CK: no mulching; SM: straw mulching. (b) Numbers followed by different lowercase letters within a row between straw mulching and no mulching are significantly different at P = 0.05 by the least square means test. (c) Numbers followed by different uppercase letters within a row between incubation temperatures are significantly different at P = 0.05 by the least square means test. (PDF) [file pone.0237245.s004.pdf]

S3 Table. Relative abundances (%) of bacterial taxa under straw mulching and no mulching after short-term incubation at different temperatures.

Phylum	Class	Order	15ºC		25ºC		35ºC	
			CK[a]	SM	CK	SM	CK	SM
Proteobacteria			26.9b[b]A[c]	30.3aA	28.5aA	30.8aA	30.0aA	34.0aA
	Gammaproteobacteria		7.34aA	8.01aA	7.84aA	8.03aA	9.16aA	12.0aA
		Xanthomonadales	1.78aAB	2.13aA	1.88aA	2.46aA	1.63aB	1.81aA
		Pseudomonadales	5.21aA	5.41aA	5.71aA	5.15aA	7.22aA	9.83aA
	Alphaproteobacteria		8.84bA	9.79aA	7.82aB	8.95aA	7.56aB	6.19bB
		Rhodospirillales	2.42bA	3.18aA	2.80bA	3.26aA	3.06aA	2.65aA
		Rhizobiales	3.21aA	3.51aA	3.08aA	3.40aA	2.56aA	1.95aB
		Sphingomonadales	2.93aA	2.73aA	1.68aB	1.89aB	1.62aB	1.28bC
	Betaproteobacteria		5.58bA	6.47aB	6.98aA	6.58aB	7.57aA	8.99aA
		Nitrosomonadales	3.81aA	3.98aB	5.27aA	4.38aB	5.99aA	6.31aA
		Burkholderiales	0.903bA	1.40aA	0.916aA	1.16aA	0.742aA	1.56aA
	Deltaproteobacteria		5.07bA	5.99aB	5.81bA	7.24aA	5.67bA	6.85aAB
		Myxococcales	3.71aA	3.63aB	4.54aA	4.56aA	4.09aA	4.53aA
		Desulfurellales	0.876bA	1.56aA	0.719bA	1.38aA	0.737aA	1.04aB
Actinobacteria			18.8aA	15.0bA	21.5aA	14.6bA	20.2aA	13.5bA
	Actinobacteria		6.86A	5.05A	9.34aA	4.73bA	8.36aA	4.323bA
		Acidimicrobiales	3.73aA	3.71aA	3.38aA	3.07aAB	3.57aA	2.45aB
		Frankiales	1.94aA	1.64aA	2.64aA	2.03aA	2.28aA	1.63aA
		Micromonosporales	1.19aA	0.916aA	2.05aA	1.17aA	1.91aA	0.775aA
	Thermoleophilia		5.92aA	4.41bA	6.32aA	3.10bA	5.81aA	4.69bA

		Solirubrobacterales	3.76aA	2.69bA	4.14aA	2.53bA	3.75aA	2.73bA
		Gaiellales	2.17aA	1.72bAB	2.18aA	1.49bB	2.05aA	1.96aA
	Acidimicrobiia		3.73aA	3.71aA	3.38aA	2.90aAB	3.57aA	2.45aB
	MB-A2-108		1.78aAB	1.39bA	1.70aB	1.33aA	1.83aA	1.59aA
Chloroflexi			17.8aA	14.2bA	16.7aA	14.3bA	13.0aB	12.8aA
	Anaerolineae		5.53aA	4.95aA	5.71aA	5.18aA	3.84aB	4.49aA
	KD4-96		2.67aA	2.01bA	2.15aAB	2.08aA	2.06aB	1.71aA
	Chloroflexia		2.16aA	1.35bA	2.46aA	1.43bA	1.70aA	1.47aA
		Chloroflexales	2.01aA	1.29bA	2.37aA	1.35bA	1.58aA	1.43aA
	Thermomicrobia		2.32aA	1.21bA	2.09aA	1.21bA	1.61aB	1.24bA
		JG30-KF-CM45	2.16aA	1.06bA	1.97aA	1.07bA	1.50aB	1.16bA
	Gitt-GS-136		1.75aA	0.966bA	1.26aA	0.700bB	1.20aA	0.577bC
Acidobacteria			15.6aA	16.5aA	13.6bA	15.9aA	13.5aA	14.1aB
	Subgroup_6		6.49aA	6.59aA	5.89bA	6.88aA	5.94aA	6.11aA
	Blastocatellia		4.27aA	4.22aA	3.40aB	3.50aB	3.54aB	3.27aB
	Holophagae		2.29bA	2.78aA	1.78bB	2.30aB	1.45aB	1.68aC
		Subgroup_10	1.03bA	1.34aA	0.818bA	1.17aB	0.783aA	0.797aC
	Subgroup_17		1.06aA	0.895aA	1.13aA	1.06aA	1.02aA	1.10aA
Gemmatimonadetes			8.97aA	8.22aAB	7.93aA	7.95aB	9.43aA	9.61aA
Nitrospirae			4.06aA	5.41aAB	4.09aA	6.21aA	3.80aA	5.00aB
Planctomycetes			3.72aA	4.86aA	3.54aA	4.90aA	3.62aA	4.94aA
	OM190		1.30bA	1.99aA	1.29aA	2.04aA	1.09aA	1.85aA
	Planctomycetacia		1.32aA	1.14aA	1.05aA	0.983aA	1.23aA	0.927aA
	Phycisphaerae		0.655bA	1.10aA	0.679bA	1.21aA	0.781bA	1.49aA
Bacteroidetes			1.45bA	1.95aA	1.11bB	1.75aAB	1.22bAB	1.36aB

a CK: no mulching; SM: straw mulching

b Numbers followed by different lowercase letters within a row between straw mulching and no mulching are significantly different at P=0.05 by the least square means test.

c Numbers followed by different uppercase letters within a row between incubation temperatures are significantly different at P=0.05 by the least square means test.
