# Supplementary material for: Increasing temperature can modify the effect of straw mulching on soil C fractions, soil respiration, and microbial community composition
Source: PLoS One. 2020 Aug 11;15(8):e0237245. doi: 10.1371/journal.pone.0237245 (PMC7418978; doi:10.1371/journal.pone.0237245)
Supplement: S4 Table — (a) CK: no mulching; SM: straw mulching. (b) Different lowercase letters indicate significant difference among different mulching methods or incubation temperatures. (c) NS, not significant. ***Significant at P≤0.001; *Significant at P≤0.05. (PDF) [file pone.0237245.s005.pdf]

S4 Table. Influence of straw mulching and incubation temperature on soil fungal phyla (%) after short-term incubation

Mulching method[a]	Incubation temperature (°C)	*Ascomycota*	*Basidiomycota*	*Zygomycota*	*Unidentified*
CK		79.4a[b]	6.54b	5.37a	2.50a
SM		71.9b	11.7a	7.44a	1.73a
	15	70.2b	8.75a	7.44a	2.22a
	25	76.1ab	10.5a	6.84a	1.68a
	35	80.7a	8.01a	4.93a	2.45a
<u>Significance</u>					
Treatment (T)		*	***	NS	NS
Temperature (ST)		*	NS	NS	NS
T×ST		NS[c]	*	NS	NS

a CK: no mulching; SM: straw mulching

b Different lowercase letters indicate significant difference among different mulching methods or incubation temperatures

c NS, not significant.

***Significant at $P \le 0.001$; *Significant at $P \le 0.05$.
