# Supplementary material for: Increasing temperature can modify the effect of straw mulching on soil C fractions, soil respiration, and microbial community composition
Source: PLoS One. 2020 Aug 11;15(8):e0237245. doi: 10.1371/journal.pone.0237245 (PMC7418978; doi:10.1371/journal.pone.0237245)
Supplement: S5 Table — (a) CK: no mulching; SM: straw mulching. (b) Numbers followed by different lowercase letters within a row between straw mulching and no mulching are significantly different at P = 0.05 by the least square means test. (c) Numbers followed by different uppercase letters within a row at different incubation temperatures are significantly different at P = 0.05 by the least square means test. (PDF) [file pone.0237245.s006.pdf]

S5 Table. Relative abundances (%) of fungal taxa under straw mulching and no mulching after short-term incubation at different temperatures.

Phylum	Class	Order	15°C		25°C		35°C	
			CK[a]	SM	CK	SM	CK	SM
Ascomycota			73.2a[b]A[c]	67.1aA	81.9aA	70.4bA	83.1aA	78.3bA
	Sordariomycetes		43.5aA	38.6aA	46.4aA	43.5aA	44.8aA	43.6aA
		Sordariales	11.7aB	15.3aB	15.4aB	14.8aB	22.6aA	28.4aA
		Hypocreales	25.1aA	15.9aA	23.4aA	21.8aA	20.0aA	13.3bA
		Xylariales	1.64aA	1.35aA	1.83aA	1.41aA	0.225aB	0.0928aB
	Incertae sedis		2.07aB	1.85aB	4.13aAB	5.43aA	6.18aA	6.55aA
	Eurotiomycetes		2.45aA	4.28aA	3.02aA	1.81aA	4.17aA	2.35aA
		Eurotiales	1.15aA	2.81aA	1.93aA	1.15aB	3.19aA	1.31aAB
	Dothideomycetes		4.19aA	6.62aA	5.50aA	3.72aB	2.35aA	1.73aC
		Pleosporales	2.43bA	5.52aA	3.69aA	3.07aB	1.87aA	1.50aB
	Pezizomycetes		0.865aA	1.11aA	1.28aA	0.333aA	0.805aA	0.543aA
	Lecanoromycetes		0.818aA	0.925aA	0.528aAB	0.480aA	0.362aB	0.480aA
Zygomycota			5.50bA	9.38aA	6.20bA	7.49aA	4.41bA	5.44aA
	Incertae sedis		5.50bA	9.38aA	6.20aA	7.49aA	4.41aA	5.44aA
		Mortierellales	5.17bA	8.02aA	5.91aA	4.69aA	4.13aA	3.74aA
Basidiomycota			4.77bA	12.7aAB	6.92bA	14.2aA	7.93A	8.09B
	Tremellomycetes		2.25aB	2.29aB	2.92aAB	3.14aAB	4.40aA	5.71aA
	Agaricomycetes		2.02bA	8.92aA	3.52bA	10.7aA	3.23A	1.87B
		Agaricales	1.33aA	3.79aA	1.79aA	4.86aA	1.05aA	1.03aA

Cantharellales	0.359bA	3.95aAB	1.25bA	5.30aA	1.84aA	0.331aB

a CK: no mulching; SM: straw mulching

b Numbers followed by different lowercase letters within a row between straw mulching and no mulching are significantly different at P=0.05 by the least square means test.

c Numbers followed by different uppercase letters within a row at different incubation temperatures are significantly different at P=0.05 by the least square means test.
