# Supplementary material for: Increasing temperature can modify the effect of straw mulching on soil C fractions, soil respiration, and microbial community composition
Source: PLoS One. 2020 Aug 11;15(8):e0237245. doi: 10.1371/journal.pone.0237245 (PMC7418978; doi:10.1371/journal.pone.0237245)
Supplement: S6 Table — SOC: soil organic carbon; PCM: potential C mineralization; MBC: microbial biomass carbon. (PDF) [file pone.0237245.s007.pdf]

S6 Table. Relationship between cumulative soil respiration and C fractions at different temperatures

Incubation temperature	Index	R^2	Significance
15 °C	SOC	0.899	<0.01
	PCM	0.731	<0.05
	MBC	0.061	>0.05
25 °C	SOC	0.937	<0.001
	PCM	0.835	<0.05
	MBC	0.219	>0.05
35 °C	SOC	0.984	<0.001
	PCM	0.202	>0.05
	MBC	0.001	>0.05

SOC: soil organic carbon; PCM: potential C mineralization; MBC: microbial biomass carbon
