# Supplementary material for: Increasing temperature can modify the effect of straw mulching on soil C fractions, soil respiration, and microbial community composition
Source: PLoS One. 2020 Aug 11;15(8):e0237245. doi: 10.1371/journal.pone.0237245 (PMC7418978; doi:10.1371/journal.pone.0237245)
Supplement: S7 Table — (a) CK: no mulching; SM: straw mulching. (b) Soil organic carbon (SOC); dissolved organic carbon (DOC); potential C mineralization (PCM); microbial biomass carbon (MBC); soil respiration (SR). (c) ** Correlation is significant at the 0.01 level; * Correlation is significant at the 0.05 level. (PDF) [file pone.0237245.s008.pdf]

S7 Table. Spearman's rank correlation coefficients (R) between microbial (i.e., bacterial and fungal) compositions and the soil C fractions and respiration at the phylum level.

Index	Microbial phyla	CK				SM			
		SOC	PCM	MBC	SR	SOC	PCM	MBC	SR
Bacteria	*Proteobacteria*	-0.547	-0.562	-0.119	0.600	-0.670*	-0.503	-0.313	0.380
	Actinobacteria	-0.325	0.471	-0.428	0.224	0.416	-0.046	0.263	-0.270
	Chloroflexi	0.754*	0.794*	0.450	-0.868**	0.621	0.415	0.411	-0.292
	Gemmatimonadetes	-0.028	-0.308	0.090	0.236	-0.482	-0.276	-0.008	0.577
	Acidobacteria	0.595	0.301	0.477	-0.536	0.849**	0.397	0.189	-0.771*
	Bacteroidetes	0.525	0.307	0.434	-0.560	0.802**	0.660	0.644	-0.734*
	Nitrospirae	0.170	-0.417	0.120	-0.074	0.346	0.497	-0.323	-0.255
	Planctomycetes	0.222	-0.463	0.210	-0.111	0.083	0.531	-0.022	-0.037
Fungi	*Ascomycota*	-0.460	-0.504	0.047	0.443	-0.607	-0.556	0.030	0.720*
	Basidiomycota	-0.400	-0.613	-0.342	0.663	0.554	0.562	-0.247	-0.555
	Zygomycota	0.251	0.384	0.097	-0.242	0.490	0.381	0.500	-0.527

a CK: no mulching; SM: straw mulching.

b Soil organic carbon (SOC); dissolved organic carbon (DOC); potential C mineralization (PCM); microbial biomass carbon (MBC); soil respiration (SR)

c ** Correlation is significant at the 0.01 level; * Correlation is significant at the 0.05 level.
