# Supplementary material for: Increasing temperature can modify the effect of straw mulching on soil C fractions, soil respiration, and microbial community composition
Source: PLoS One. 2020 Aug 11;15(8):e0237245. doi: 10.1371/journal.pone.0237245 (PMC7418978; doi:10.1371/journal.pone.0237245)
Supplement: S8 Table — (a) CK: no mulching; SM: straw mulching. (b) Soil organic carbon (SOC); dissolved organic carbon (DOC); potential C mineralization (PCM); microbial biomass carbon (MBC); soil respiration (SR). (c) ** Correlation is significant at the 0.01 level; * Correlation is significant at the 0.05 level. (PDF) [file pone.0237245.s009.pdf]

S8 Table. Spearman's rank correlation coefficients (R) between microbial (i.e., bacterial and fungal) composition and the soil C fractions and respiration at the class level.

Index	Microbial class	CK				SM			
		SOC	PCM	MBC	SR	SOC	PCM	MBC	SR
Bacteria	*Gammaproteobacteria*	-0.524	-0.131	-0.115	0.542	-0.594	-0.613	-0.297	0.311
	Alphaproteobacteria	0.402	0.634	0.204	-0.730*	0.909**	0.639	0.364	-0.856**
	Gemmatimonadetes	-0.141	-0.297	-0.056	0.402	-0.509	-0.398	0.058	0.597
	Betaproteobacteria	-0.414	-0.777*	-0.170	0.488	-0.587	-0.084	-0.144	0.644
	Actinobacteria	-0.464	0.282	-0.539	0.310	0.284	0.240	0.191	-0.163
	Deltaproteobacteria	-0.069	-0.326	0.075	0.424	-0.366	0.155	-0.179	0.530
	Subgroup_6	0.400	0.232	0.212	-0.296	0.554	0.281	0.101	-0.321
	Anaerolineae	0.680*	0.696*	0.309	-0.768*	0.497	0.574	0.605	-0.156
	Thermoleophilia	0.087	0.436	0.079	-0.172	-0.164	-0.697*	0.062	0.240
	Nitrospira	0.170	-0.417	0.120	-0.074	0.346	0.497	-0.323	-0.255
	Acidimicrobiia	0.146	0.571	0.001	-0.030	0.916**	0.416	0.356	-0.849**
	Gitt-GS-136	0.426	0.834**	0.269	-0.527	0.964**	0.438	0.584	-0.901**
	Thermomicrobia	0.815**	0.823**	0.457	-0.850**	0.163	-0.155	0.325	0.165
	Blastocatellia	0.486	0.481	0.468	-0.575	0.729*	0.119	0.270	-0.827**
	KD4-96	0.620	0.821**	0.420	-0.661	0.615	0.275	-0.021	-0.437
	Holophagae	0.786*	0.397	0.674*	-0.861**	0.915**	0.566	0.363	-0.965**
	OM190	0.509	-0.265	0.394	-0.321	0.252	0.585	0.119	-0.197
	MB-A2-108	-0.403	0.071	-0.256	0.381	-0.284	-0.782*	-0.136	0.385
	Phycisphaerae	-0.406	-0.748*	-0.138	0.505	-0.334	0.122	-0.180	0.448

	Chloroflexia	0.157	0.583	-0.020	-0.406	-0.143	-0.193	-0.207	0.214
	Subgroup_17	0.540	-0.044	0.470	-0.178	-0.520	-0.395	-0.251	0.723*
	Planctomycetacia	0.084	-0.040	-0.013	-0.170	0.507	0.554	0.158	-0.465
Fungi	*Sordariomycetes*	-0.159	-0.374	0.121	0.051	-0.278	-0.357	0.072	0.376
	Incertae sedis	-0.666	-0.641	-0.388	0.720*	-0.876**	-0.245	-0.546	0.902**
	Eurotiomycetes	-0.362	-0.143	-0.402	0.540	0.498	0.037	0.399	-0.585
	Agaricomycetes	-0.137	-0.409	-0.130	0.293	0.655	0.610	-0.166	-0.616
	Dothideomycetes	0.488	0.480	0.294	-0.414	0.828**	0.413	0.387	-0.959**
	Tremellomycetes	-0.569	-0.619	-0.369	0.808**	-0.794*	-0.511	-0.153	0.762*
	Pezizomycetes	-0.178	0.355	-0.381	-0.046	0.466	0.444	0.275	-0.591
	Lecanoromycetes	0.930**	0.270	0.827**	-0.798**	0.367	-0.031	0.065	-0.345

a CK: no mulching; SM: straw mulching.

b Soil organic carbon (SOC); dissolved organic carbon (DOC); potential C mineralization (PCM); microbial biomass carbon (MBC); soil respiration (SR)

c ** Correlation is significant at the 0.01 level; * Correlation is significant at the 0.05 level.
