# Supplementary material for: Increasing temperature can modify the effect of straw mulching on soil C fractions, soil respiration, and microbial community composition
Source: PLoS One. 2020 Aug 11;15(8):e0237245. doi: 10.1371/journal.pone.0237245 (PMC7418978; doi:10.1371/journal.pone.0237245)
Supplement: Attachment — Submitted filename: Response to Reviewers.docx [file pone.0237245.s010.docx]

Dear Editor and reviewers, 
We are grateful for the very helpful comments and suggestions from the editor and the reviewers. Based on the comments and suggestions, we have carefully revised our manuscript; the modified portions are marked in the revised manuscript. Our point-by- point responses to each reviewer comment are as follows.

Yours Sincerely

Xin Fu, Jun Wang, Mengyi Xie, Fazhu Zhao, Russell Doughty.

**Responses to Editor**

1. Please ensure that your manuscript meets PLOS ONE's style requirements, including those for file naming. The PLOS ONE style templates can be found at <https://journals.plos.org/plosone/s/file?id=wjVg/PLOSOne_formatting_sample_main_body.pdf> and <https://journals.plos.org/plosone/s/file?id=ba62/PLOSOne_formatting_sample_title_authors_affiliations.pdf>

**Corrected according to the templates.**

2. We noticed you have some minor occurrence of overlapping text with previous publications, which needs to be addressed. In your revision ensure you cite all your sources (including your own works), and quote or rephrase any duplicated text outside the methods section. Further consideration is dependent on these concerns being addressed.

**Corrected as suggested. References have been added and relative descriptions have been corrected (Lines 192-206, 234-241 in the Revised Manuscript with Track Changes).**

3. We suggest you thoroughly copyedit your manuscript for language usage, spelling, and grammar. If you do not know anyone who can help you do this, you may wish to consider employing a professional scientific editing service.

**We asked Dr. Russell Doughty, who is working in Division of Geological and Planetary Sciences, California Institute of Technology, Pasadena, CA, USA, for language improvements. Also, he is included in the authors’ list in the revised manuscript.**

4. We note that you are reporting an analysis of a microarray, next-generation sequencing, or deep sequencing data set. PLOS requires that authors comply with field-specific standards for preparation, recording, and deposition of data in repositories appropriate to their field. Please upload these data to a stable, public repository (such as ArrayExpress, Gene Expression Omnibus (GEO), DNA Data Bank of Japan (DDBJ), NCBI GenBank, NCBI Sequence Read Archive, or EMBL Nucleotide Sequence Database (ENA)). In your revised cover letter, please provide the relevant accession numbers that may be used to access these data. For a full list of recommended repositories, see http://journals.plos.org/plosone/s/data-availability#loc-omics or <http://journals.plos.org/plosone/s/data-availability#loc-sequencing>.

**The raw sequences for all samples were sent to the Sequence Read Archive (SRA) database of the National Center for Biotechnology Information (NCBI, Bethesda, MD, USA) with the accession numbers SRP260944 for bacteria and SRP261054 for fungi, respectively.**

5. Please provide further details in your Methods section on the sequencing carried out on the samples.

**Changed. See Lines 242-268.**

6. PLOS requires an ORCID iD for the corresponding author in Editorial Manager on papers submitted after December 6th, 2016. Please ensure that you have an ORCID iD and that it is validated in Editorial Manager. To do this, go to ‘Update my Information’ (in the upper left-hand corner of the main menu), and click on the Fetch/Validate link next to the ORCID field. This will take you to the ORCID site and allow you to create a new iD or authenticate a pre-existing iD in Editorial Manager. Please see the following video for instructions on linking an ORCID iD to your Editorial Manager account: <https://www.youtube.com/watch?v=_xcclfuvtxQ>

**Done.**

7. Please upload a copy of Figures 7 and 8, to which you refer in your text on page xx. If the figure is no longer to be included as part of the submission please remove all reference to it within the text.

**Removed. We rechecked this sentence, and the description about Figures 7 and 8 is unnecessary. See Line 407.**

8. Please include captions for your Supporting Information files at the end of your manuscript, and update any in-text citations to match accordingly. Please see our Supporting Information guidelines for more information: http://journals.plos.org/plosone/s/supporting-information.

**Changed. We have revised Supporting Information according to the guidelines. (Lines 574-623)**

**Responses to Reviewer #1**

Line 1: How can soil C fraction, soil respiration, and community composition have responses? E The title is misleading. Please restructure the sentence.

**The title was changed as “***Increasing temperature can modify the effect of straw mulching on soil C fractions, soil respiration, and microbial community composition***” (Lines 1-2 in the Revised Manuscript with Track Changes).**

Line 19: what mulched?

**“*what*” was deleted (Line 22).**

Line 21-22: Restructure sentence, reads weird.

**The sentence was corrected as “***Soil respiration, C fractions and bacterial and fungal community structure were measured.***” (Lines 24-25).**

Line 22: Greater than what?

**The sentence was corrected as “***SM had greater soil organic carbon and potential C mineralization and a similar microbial biomass carbon throughout the incubation when compared with CK***” (Line 26-28).**

Line 23: SM was averaged across temperatures?

**Deleted. (Line 28)**

Line 24: Greater than what?

Line 25: Again, greater than what?

**The sentences have been corrected as “***Similar microbial community composition was found in the soils with SM and CK before incubation. However, SM had a greater bacterial richness and the relative abundances of Proteobacteria, Acidobacteria, Nitrospirae, Planctomycetes, Bacteroidetes, and Basidiomycota, but a lower relative abundance of Actinobacteria, Chloroflexi, and Ascomycota than CK after incubation***” (Lines 31-35).**

Line31: What is the existing relationship that was modified with increasing temperature?

**This sentence was corrected as “***These findings indicated that the effect of straw mulching on soil C cycling and microbial community structure can be highly modified by increasing temperature*.**” (Lines 41-43).**

Line 33: The authors haven’t really looked at carbon pools nor microbial activities, so this is not an appropriate keyword

**The keywords were corrected as “***Incubation, mulching, microbes, soil carbon, warming***” (Line 44).**

Line 35: Numbers are available for this, please write the percentages instead of writing a vague word as “substantially”

**This sentence was corrected as “***Soil contains around 1500 Pg of organic carbon (C) and plays a major role in the carbon cycling in terrestrial ecosystems***” (Lines 47-48).**

Line 36: What is meant by small shift? And the sentence structure is wrong.

**This sentence was corrected as “***A small variation in soil C sequestration can lead to a significant change in atmospheric CO_2_ concentration***” (Lines 49-50).**

Line 39-40: “SR is often used to approximate the rate of soil organic carbon (SOC) mineralization and decomposition” Please provide reference. Is this true for all soil orders and latitudes? Avoid making such general statements. I would recommend refer to these and references cited in these papers to develop a deeper understanding on this topic and improve the introduction. <https://agupubs.onlinelibrary.wiley.com/doi/pdf/10.1002/2017GB005644,> <https://www.biogeosciences.net/16/663/2019/,> [https://www.nature.com/articles/srep18370](https://www.nature.com/articles/srep18370)

(1) **The sentence was deleted and reviewed. (Line 86-87)**
(2) **The papers of Wei et al. (2015) and Meyer et al. (2018) were cited in the manuscript.**

Line42-43: This statement makes no sense. If the discussion has rarely been limited, why do this study. Also, which species are the authors alluding to here? Vague sentence.

**The sentence was deleted.**

Line 46: changing how and promoting what soil microbial processes?

**Deleted.**

Line 48: if previous studies have already studied this, why are the authors doing this experiment? What is unique?

**Though previous studies have already studied the responses of SOC, SR and microbial activity to warming have gained more attentions recently. The comprehensive responses of C soil fractions, SR and microbial communities to straw mulching would vary with different temperature change is now well reported. (Lines 71-73)**

Lines 52-54: dryland carbon dynamics is a new concept introduced here, why?

**we have deleted the concept of “dryland carbon dynamics”; the straw mulching was widely application in the dryland, so we used the concept, but the concept of “dryland carbon dynamics” in manuscript was not inaccurate.**

Line 69: why study soil C fractions? The authors haven’t presented a compelling reason.

**We have added the reason why study soil C fractions as “***Generally, SOC would change slowly with management practices due to its large pool sizes and inherent spatial variability. Soil labile C fractions, such as microbial biomass carbon (MBC) and potential C mineralization (PCM) would response more rapidly to environmental change than SOC.***” (Lines 56-59).**

Line 72: The authors should provide some background information about mulching practices and why there is a need to study it. What have previous studies reported? Have other studied looked at impact of variable temperature on mulching benefits?

**Reviewed. The background information about mulching practices and the previous results about temperature on mulching have added as “***In the recent decades, straw mulching has widely been adopted to conserve soil water, regulate soil temperature and increase crop yield in dryland cropping systems. The application of straw mulch also has been proposed as a method to store organic carbon long term. In an 8-yr study in the Loess Plateau of China, Wang et al. reported that soil organic C (SOC) stock was 7-35% greater with straw mulching than without***”.** (**Lines 51-56)**

Line 73: What does one mean by changing the relationships?

**Changed. We have revised the sentence from “***explore whether temperature affected soil C sequestration by changing the relationships among soil respiration, microorganisms, and C fractions***” to “***In the context of climate change, the responses of SOC, SR and microbial activity to warming have gained more attentions recently. Increasing temperature would stimulate soil microbial metabolisms, accelerate SOC decomposition and increase C efflux through SR. Since the temperature sensitivity of SR (Q_10_) varied with the substrate availability, how the comprehensive responses of C soil fractions, SR and microbial communities to straw mulching would vary with different temperature change is now well reported.***”. (Lines 71-76)**

Line 71-74: What hypotheses are driving these objectives?

**We have added two hypotheses as “***(1) straw mulching would increase SR rates by increasing the input of organic matter in the soil compared with no mulching; and (2) straw mulching could change the temperature sensitivity of the agro-ecosystem due to the regulation of soil C fractions and soil microbial community.***”. (Lines 80-83).**

Line 134: Where are the codes for sequence analyses? Where have the sequences been deposited? Please make code and data available.

**Added. The raw sequences for all samples have sent to the Sequence Read Archive (SRA) database of the National Center for Biotechnology Information (NCBI, Bethesda, MD, USA) under the accession numbers SRP260944 and SRP261054 for bacteria and fungi, respectively. (Line 268)**

Line 194: OTU composition

**Changed. “***OUT***” was changed to “***OTU***”. (Line 365).**

Line 224-244 and Figure 5: Are these averages? How were the three replicates handled? It is unclear whether these relative abundances are from one replicate per treatment or average across replicates.

**Figure 5 data showed the average across three replicates.**

Line 319-320: taxa did not differ due to low microbial activity is a stretch. The environment is not significantly different enough to see a difference

**The sentence “***which is probably due to lower microbial activity***” was removed. (Line 516).**

Line 324: What does one mean by “separated by PCA”?

**We have revised the sentence as “***According to PCA analysis, bacterial and fungal communities were significantly different from each other with and without straw mulch and for the incubation temperatures***”. (Lines 519-520).**

Line 324-325: I would recommend that the authors refer to studies already conducted to understand the effect of straw mulching on bacteria. <https://www.sciencedirect.com/science/article/pii/S092913931931056X#f0030>
[https://link.springer.com/article/10.1007/s13762-017-1434-8,](https://link.springer.com/article/10.1007/s13762-017-1434-8,)
<https://www.sciencedirect.com/science/article/pii/S1164556318304874>
**Added. We have added the references of Fu et al. (2019); Huang et al. (2019); Wang et al. (2020).**

Figure3: How were these ellipses drawn?

**These ellipses represent significant differences between different treatments. The purpose of these ellipses is making readers see the difference of two treatments more clearly. The method was refered to Hemkemeyer et al. (2015).**

**Responses to Reviewer #2**

Introduction
Line 43 Perhaps “defined” instead of “limited”.

**Agree. We have deleted the sentence. (Line 123 in the Revised Manuscript with Track Changes)**

The introduction focuses primarily on carbon availability and temperature as strong controls on soil respiration in general. However, the focus on dryland systems is cursory at best (line 53-54). Please include additional details about how this information is important for dryland systems.

**We have added the description about the development of straw mulching in dryland. (Lines 51-56)**

Results
Line 167-168: It was stated that cumulative respiration was greater in SM than CK but only 3 values were provided for cumulative respiration at the different temperatures. To which treatment do these three values apply? Throughout the results there are figure captions listed. (line 172-186, 201-204)

**Each value is an average of three replicates for every treatment. We have added figs captions. (Lines 239, 243-245, 253, 284, 288 and 306)**

Discussion
The authors discuss changes in respiration at temperature, but there is little reference to the temperature sensitivity calculations they performed. Additionally, was temperature sensitivity calculated at different points throughout the experiment, only 1 value per treatment was included. Additional temperature sensitivity calculations would enable additional comparisons to other studies and provide a standard metric for comparison.

**We have added the discussion about temperature sensitivity and compared with other studies as “** *The result by Wang et al. showed that Q_10_ values in all soils ranged from 1.96 to 2.76, which was higher than our study. This was probably due to different soil environments (soil organic matter, moisture, microorganism, temperature) compared with our study. In the recent decade, some incubation studies illustrated that soils with high C substrate quality have low Q_10_, which was consistent with our study (Table 3). In our present study, straw mulching had lower Q_10_ as compared with no mulching. Higher soil microorganisms under straw mulching may accelerate the consumption of C directly caused by soil respiration. But no additional C sources were added during the incubation experiment, causing soil C is insufficient at the late incubation stage, and resulting in the decrease of the Q_10_ under the straw mulching. Similarly, lower qCO_2_ under straw mulching also confirmed these conclusions (Fig 3). However, in our previous study, we found that the Q_10_ was higher under straw mulching than no mulching, probably due to the driver factors was different as the present study. Dai et al. also indicated that the temperature sensitivity of organic C mineralization in the exogenous C is highly sensitive to SOC, compared with no C input.***”. (Lines 495-512)**

Lines 273-294: The authors describe that it is possible the microorganisms have reduced respiration relative to biomass at lower temperatures, however that calculation does not appear in the manuscript. Respiration per unit biomass (sometimes referred to as Rmass) would be a helpful metric for comparison between temperature and treatments as well as other studies. I think that the authors have MBC data and respiration data for at least some of the time points for this calculation.

**We have added the description about metabolic quotient (*q*CO_2_), and found “** *The qCO_2_ under both CK and SM appeared to increase with increasing incubation temperatures (Fig 3). For all incubation temperatures, SM had higher qCO_2_ than CK*.**”. (Lines 322-324 and Fig 3.)**

Tables and Figures
Figure 1b. Is this graph showing an average soil respiration for CK and SM at each temperature or is this only one of the treatments? If one of the treatments then needs to be labeled as such in the caption. If it is an average of the two treatments I don’t think they can be combined since there was a difference in respiration based on treatment.

**It is an average of the two treatments, yes, there was a difference in respiration based on different treatments. We are agreed with this view and have revised the figure, which showed Line 326**

Table 2. Between what temperatures was Q10 calculated, 15 to 25, 25 to 35, or 15 to 35?
**Q_10_ was calculated from 15 to 25, 25 to 35. We have added the formulas and reference. (Lines 282-289)**

**Responses to Reviewer #3**

However, I have certain and major concern about Soil C pools and how the authors relate that to microbial habitat, besides, what was the real decomposition process and how was related to increasing temperature. There is lack of information about straw mulch in terms of composition and C: N Ratio.

**Reviewed (1) The manuscript mainly focuses on the response of carbon pools under straw mulching to global warming. Therefore, we mainly analyzed the influence of straw mulching on soil C fractions, SR rate and soil microbial community.**

**(2) The information about straw mulch in term of composition and C/R ratio was added as “***Corn straw had a C/N ratio of 40.1 and the contents of cellulose, hemicellulose and lignin were 32%, 28% and 15.5%, respectively.***”. (Lines 197-198).**

Other comments:
-Abbreviations in the text are confusing and need to be more clear

**Reviewed.**

-Heading, subheading, subtitles in the manuscript need to be changed to appropriate format.

**Changed.**

-Objectives are not clear, and the second objective is part of the first one. Basically, the study has more than one objective.

**Objectives were revised as “***(1) determine changes in carbon fractions and SR rates to different temperatures in soils with and without straw mulch, (2) quantify the effect of straw mulch on soil temperature sensitivity; (3) explore the relationships among soil C fractions, SR rates and soil microbial community in a wheat cropping system in the Loess Plateau, China***” (Lines 90-94).**

-There is no hypothesis related to the objectives to show the mulching impact and warming as a drive factor for decomposition and microbial activity.

**Two hypothesis were added as “***(1) determine changes in carbon fractions and SR rates to different temperatures in soils with and without 9-yr straw mulching, (2) quantify the effect of straw mulching on soil temperature sensitivity; and (3) explore the relationships among soil C fractions, SR rates and the soil microbial community.***”. (Lines 77-80).**

-Separate table for initial chemical and physical soil properties is needed and it is better than the way was written in the text.

**The tables about soil C fractions was revised. We have separated the data of initial chemical and physical soil properties from after incubation. See Tables 1 and 4.**

-Lack of information about mulch application

**The information about mulch application was added as “***In the recent decades, straw mulching has widely been adopted to conserve soil water, regulate soil temperature and increase crop yield in dryland cropping systems. The application of straw mulch also has been proposed as a method to store organic carbon long term.***” (Lines 51-54).**

-Tables need to be revised

**Reviewed.** **See Tables 1, 2, 4 and 5.**

-Line 41 – 47: the statement is not clear and needs to be revised

**The descriptions about the development of temperature on SR and microbial community was revised as “***A comprehensive understanding of how soil respiration is affected by changing different factors is important to improving soil carbon sequestration. However, positive, neutral, and negative effects of temperature on soil C fractions and SR have been documented. Thus the temperature dependency of soil C decomposition and its possible future variation needs to be more clearly defined.***” and “***In the context of climate change, the responses of SOC, SR and microbial activity to warming have gained more attentions recently. Increasing temperature would stimulate soil microbial metabolisms, accelerate SOC decomposition and increase C efflux through SR*.**”. (Lines 71-73)**

-Line 82: what is STN stands for?

**We have changed “*STN*” to “*soil total nitrogen content*”. (Line 190)**

-Line 95-98 need more details about soil sampling,

**The details about soil sampling was added as “***Fresh soil samples were collected from after corn harvest in October 2017. Soil samples (about 10 kg) were collected with a spade from the surface layer (0-20 cm) from five places within a plot. Then we composited five samples to one sample, and placed them in plastic boxes. We tried to avoid damaging the soil structure during the collection process.***”. (Lines 207-211)**

-Line 103: In what basis you adjust soil sample to 60% water.

**The cumulative soil respiration is maximum when the water content reached 60% of the field water holding capacity. Therefore, during the incubation period, we used weighing method to keep the soil water content. The citation was added in Line 218.**

-Line 110-115: need citation

**The citation was added. See Lines 221.**

-Line 128-132: need to be revised

**The** **methods section on the sequencing was revised as “***Soil DNA was extracted from 0.5 g of freeze-dried soil using Fast DNA SPIN extraction kits (MP Biomedicals, Santa Ana, CA, USA). The extraction method was same as Ren et al. The universal Eubacterial primers 338F (5’-ACTCCTACGGGAGGCAGCA) and 806R (5’-GGACTACHVGGGTWTCTAAT-3’) were used for amplifying the 16S rRNA V3-V4 fragment. The universal eukaryotic primers ITS5F (5’-GGAAGTAAAAGTCGTAACAAGG) and ITS1R (GCTGCGTTCTTCATCGATGC) were used for amplifying the ITS-1 region.***”.** **(Lines 154-159)**

-Line 207-208 the dominant bacterial phyla total composition doesn’t come out to 100%

**The dominant bacterial phyla total composition count for about 95%, and the other bacterial phyla count for about 5%. See Line 378.**

-Line 307-308 how and need citation

**The need citation was added as “***Soil organic matter may be stabilized in the form of organo-mineral complexes, which are resistant to microbial degradation.***”. (Lines 482-483)**
